# Effect of leaflet-to-chordae contact interaction on computational mitral valve evaluation

**DOI:** 10.1186/1475-925X-13-31

**Published:** 2014-03-20

**Authors:** Yonghoon Rim, David D McPherson, Hyunggun Kim

**Affiliations:** 1 Division of Cardiovascular Medicine, Department of Internal Medicine, The University of Texas Health Science Center at Houston, Houston, TX 77030, USA

**Keywords:** Mitral valve, Finite element, Contact interaction, Coaptation, Echocardiography

## Abstract

**Background:**

Computational simulation using numerical analysis methods can help to assess the complex biomechanical and functional characteristics of the mitral valve (MV) apparatus. It is important to correctly determine physical contact interaction between the MV apparatus components during computational MV evaluation. We hypothesize that leaflet-to-chordae contact interaction plays an important role in computational MV evaluation, specifically in quantitating the degree of leaflet coaptation directly related to the severity of mitral regurgitation (MR). In this study, we have performed dynamic finite element simulations of MV function with and without leaflet-to-chordae contact interaction, and determined the effect of leaflet-to-chordae contact interaction on the computational MV evaluation.

**Methods:**

Computational virtual MV models were created using the MV geometric data in a patient with normal MV without MR and another with pathologic MV with MR obtained from 3D echocardiography. Computational MV simulation with full contact interaction was specified to incorporate entire physically available contact interactions between the leaflets and chordae tendineae. Computational MV simulation without leaflet-to-chordae contact interaction was specified by defining the anterior and posterior leaflets as the only contact inclusion.

**Results:**

Without leaflet-to-chordae contact interaction, the computational MV simulations demonstrated physically unrealistic contact interactions between the leaflets and chordae. With leaflet-to-chordae contact interaction, the anterior marginal chordae retained the proper contact with the posterior leaflet during the entire systole. The size of the non-contact region in the simulation with leaflet-to-chordae contact interaction was much larger than for the simulation with only leaflet-to-leaflet contact.

**Conclusions:**

We have successfully demonstrated the effect of leaflet-to-chordae contact interaction on determining leaflet coaptation in computational dynamic MV evaluation. We found that physically realistic contact interactions between the leaflets and chordae should be considered to accurately quantitate leaflet coaptation for MV simulation. Computational evaluation of MV function that allows precise quantitation of leaflet coaptation has great potential to better quantitate the severity of MR.

## Background

The mitral valve (MV) has the most complex structural characteristics of the four human heart valves [1-3]. All components of the MV apparatus (mitral annulus, anterior and posterior leaflets, chordae tendineae, and papillary muscles) have very complicated interrelations that work together to provide the optimized valvular function. Abnormalities in any of these components may lead to valvular dysfunction: the most common pathologies include prolapse, regurgitation, annular dilation, and chordal rupture. In regurgitation, a well-coordinated action of the interrelated components of the MV apparatus plays an important role in preventing systolic backflow of blood into the left atrium [4].

Advances in ultrasound imaging techniques have allowed clinicians to accurately assess MV physiology, and have aided in determining the degree of valvular abnormalities and the necessity for appropriate surgical intervention. Conventional two-dimensional (2D) echocardiography provides excellent insight into the complex cardiac anatomy in real time [5]. Three-dimensional (3D) echocardiography provides further detailed morphology of the MV apparatus better contributing to our understanding of the anatomical and physiologic characteristics of MV function [6]. However, biomechanical information (e.g., high stress concentration, abnormal strain distribution, malformed leaflet bending deformation, etc.) over the MV structure cannot be obtained from echocardiography. It is well known that mechanical stress is one of the primary triggering factors in deterioration and failure of heart valve tissue [7-10]. Computational simulation using numerical analysis methods can help to assess the complex biomechanical and functional characteristics of the MV apparatus [8,9,11-16]. Finite element (FE) analysis has been employed to determine deformation patterns and stress distributions across heart valve tissue under the physiologic conditions [11].

Using FE analysis and computational simulation, it is important to correctly model the contact interaction between the MV apparatus components. We have recently demonstrated that failure of proper leaflet coaptation leads to reversal of blood flow from the left ventricle into the left atrium, i.e., mitral regurgitation (MR) [17,18]. Contact interaction between the two MV leaflets is generally modeled by surface-to-surface contact such that two master and slave pairs are enforced by the penalty constraint to characterize appropriate contact interaction between the soft and wet leaflet surfaces [8,15,16,19-21]. There is an additional type of primary physical contact interaction between the MV apparatus components: leaflet-to-chordae contact interaction. A proper computational modeling strategy for this contact interaction between the leaflets and chordae tendineae is required to more accurately perform simulations of MV function. Previous computational MV studies focused primarily on the contact interaction between the MV leaflets as an interface pair [8,15,16,19-21]. However, the effect of leaflet-to-chordae contact interaction on computational MV evaluation has not been fully investigated.

We hypothesize that leaflet-to-chordae contact interaction plays an important role in computational MV evaluation, specifically in quantitating the degree of leaflet coaptation directly related to the severity of MR.

In this study, we have acquired two different MV types from patients using 3D echocardiographic data, modeled virtual MVs, performed dynamic FE simulations of MV function with and without incorporation of leaflet-to-chordae contact interaction, and determined the effect of leaflet-to-chordae contact interaction on the computational MV evaluation.

## Methods

### Virtual MV modeling

The Committee for the Protection of Human Subjects at The University of Texas Health Science Center at Houston approved our translational research protocol to utilize 3D transesophageal echocardiography (TEE) for computational modeling of patient MVs. A Philips iE33 ultrasound system (Philips Medical Systems, Bothell, WA) with a 3D TEE transducer was utilized for imaging of the patient MV apparatus. Following our standard clinical protocols, 3D TEE was performed to acquire the MV geometric data in two patients. One patient had a normal MV without MR, and another had a pathologic MV with MR. MV modeling was conducted by our virtual MV modeling protocol [17,18]. Briefly, the geometric information of the MV apparatus including annulus, leaflets and papillary muscles at end diastole (open) was identified from 3D TEE data, segmented, and traced in the cylindrical coordinate system using a custom-designed semi-automated image processing algorithm. The geometric data was transformed into the Cartesian coordinate system, followed by virtual MV modeling. The mitral leaflets and annulus at end diastole were created utilizing the non-uniform rational B-spline (NURBS) surface modeling technique, and meshed with 3D triangular shell elements. The chordae tendineae were modeled by adding 3D line (truss) elements between the papillary muscles and the leaflet edge.

### Material modeling and boundary conditions

Detailed protocols of material modeling and dynamic FE simulation of MV function are described in a previous study [18]. The MV leaflet tissue was modeled as an anisotropic hyperelastic material using a general Fung-type elastic constitutive model. The stress–strain relationship of the leaflet tissue was defined along the circumferential and radial directions. Material parameters were determined by fitting the constitutive model to the biaxial mechanical test data of the anterior and posterior leaflet tissue from a previous study [22]. These experimentally determined material characteristics data of the anterior and posterior leaflet tissue were implemented into the Fung-type elastic material model in ABAQUS (SIMULIA, Providence, RI). Leaflet thickness was set to be 0.69 mm and 0.51 mm for the anterior and posterior leaflets, respectively [23].

The chordae tendineae were modeled as nonlinear hyperelastic materials utilizing the first and second order Ogden models. Experimentally determined material characteristics of the posterior marginal chordae, anterior marginal chordae, and strut chordae from a previous study [19] were incorporated into our MV modeling, respectively. Cross-sectional areas were set 0.27 mm^2^ for the posterior marginal chordae, 0.29 mm^2^ for the anterior marginal chordae, and 0.61 mm^2^ for the strut chordae [19]. Density and Poisson’s ratio of the tissue were set at 1,100 kg/m^3^ and 0.48, respectively [14,21].

Mitral annulus and papillary muscle configurations at end diastole (open) and peak systole (closed) were pre-determined in the 3D TEE data. Combined with time-varying nonlinear nodal displacements between end diastole and peak systole, dynamic boundary motion was defined for both the annulus and the papillary muscles [18]. Free rotational movement was allowed at the annulus and papillary muscle tips. Time-varying physiologic transvalvular pressure loading was applied onto the leaflets for dynamic simulation of MV function across the cardiac cycle.

### Contact modeling

The contact interaction in computational MV simulations primarily consists of leaflet-to-leaflet and leaflet-to-chordae contacts. In ABAQUS, the general contact algorithm allows to define edge-into-edge contact penetrations [24]. The general contact algorithm creates contact forces to resist edge-into-edge penetration (i.e., interaction between the shell perimeter edges and/or the chordae truss edges). The penalty method is used to enforce contact constraints in the general contact algorithm. The penalty contact algorithm searches for slave node penetrations and edge-to-edge penetrations, and equal and opposite forces are applied to the slave nodes to oppose the penetration [24]. This process involves the addition of penalty terms in FE analysis.

**Figure 1:**
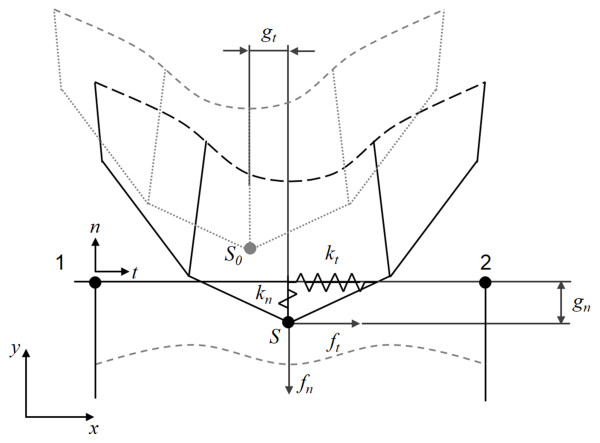
A schematic of a contact pair (slave and master surfaces) and variables used in the penalty method.

Figure 1 demonstrates a contact pair with frictional contact, which has a slave node (point *S*) and a master line (connecting node 1 and 2) in the general contact algorithm. *S*_*0*_ and *S* refer to the slave node before and after loading, respectively [25]. The penalty method utilizes the penalty term which contains the predetermined stiffness matrix (penalty stiffness) of the contact surface. The stiffness matrix of the contact surface is incorporated into the stiffness matrix of the contacting body:

(1) $$\left[K_{b}+K_{c}\right]u=F$$

where *K*_*b*_ is the stiffness matrix of contacting body, *K*_*c*_ is the stiffness matrix of contact surface, *u* is the displacement, and *F* is the applied force [25]. Any violation of the contact condition is punished by increase of the total virtual work by the penalty term. The first variation of the potential energy of the contact element is

(2) $$\delta {П}_{c}=f_{n}\delta g_{n}+f_{t}\delta g_{t}=k_{n}g_{n}\delta g_{n}+k_{t}g_{t}\delta g_{t}$$

where *k*_*n*_ is the penalty term along the normal direction, *k*_*t*_ is the penalty term along the tangential direction, and *g*_*n*_ and *g*_*t*_ are the penetration and sliding along the normal and tangential directions, respectively [25]. Hence, the equation can be written as follows:

(3) $$f_{n}\delta g_{n}+f_{t}\delta g_{t}=k_{n}g_{n}\delta g_{n}+\mathrm{sgn}\left(g_{t}\right)\mu_{d}k_{n}g_{n}\delta g_{t}$$

(4) $$f_{n}=k_{n}g_{n}$$

(5) $$f_{t}=-\mathrm{sgn}\left(g_{t}\right)\mu_{d}\left(k_{n}g_{n}\right)$$

(6) $$\mathrm{sgn}\left(g_{t}\right)=\left\{\begin{array}{c} \begin{array}{cc} -1 & \mathrm{for}\;g_{t}<0 \end{array}\\ \begin{array}{cc} 0 & \;\mathrm{for}\;g_{t}=0 \end{array}\\ \begin{array}{cc} 1 & \;\mathrm{for}\;g_{t}>0 \end{array} \end{array}\right)$$

with *μ*_*d*_: a coefficient of kinetic friction.

**Figure 2:**
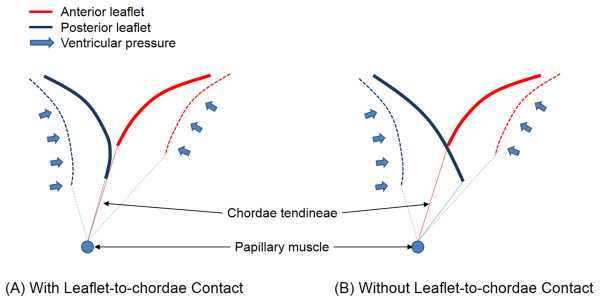
**Contact configurations between the MV apparatus components with and without consideration of leaflet**-**to**-**chordae contact interaction.**

A schematic of the MV contact configuration under the normal physiologic conditions is displayed in Figure 2A. Physiologically appropriate leaflet-to-leaflet and leaflet-to-chordae contacts during systole are demonstrated. If FE analysis employs leaflet-to-leaflet contact alone (i.e., without incorporation of leaflet-to-chordae contact), penetration of the chordae tendineae into the leaflet structure is expected during the entire systolic phase (Figure 2B).

Computational MV simulation with full contact interaction was specified such that the contact domain of the general contact algorithm was defined to incorporate entire available contact interactions between the leaflets and chordae tendineae. Computational MV simulation without leaflet-to-chordae contact interaction was specified by defining the anterior and posterior leaflets as the only contact inclusion in the general contact domain. A frictional coefficient value of 0.05 was utilized to describe the frictional behavior between the contact tissue [20].

## Results

### MV leaflet deformation

**Figure 3:**
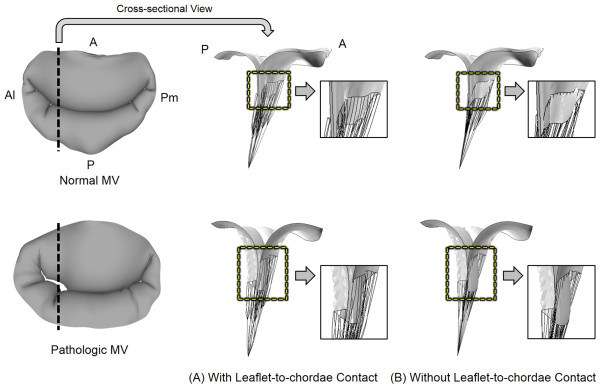
Leaflet deformation at peak systole with and without incorporation of leaflet-to-chordae contact interaction.

Figure 3 exhibits leaflet deformation at peak systole with and without incorporation of leaflet-to-chordae contact interaction. The difference in leaflet deformation between the two contact modeling methods was best demonstrated in the vicinity of the commissural regions. The posterior leaflets of both normal and pathologic MVs were longer than the anterior leaflet in the commissural regions. With leaflet-to-chordae contact interaction incorporated, the anterior marginal chordae retained the proper contact with the posterior leaflet during the entire systole (Figure 3A). On the other hand, the anterior marginal chordae demonstrated unrealistic penetration into the posterior leaflet during systole without leaflet-to-chordae contact interaction incorporated (Figure 3B). In particular, the pathologic MV with MR demonstrated a greater degree of chordal penetration in the anterolateral commissural region compared to the normal MV.

**Figure 4:**
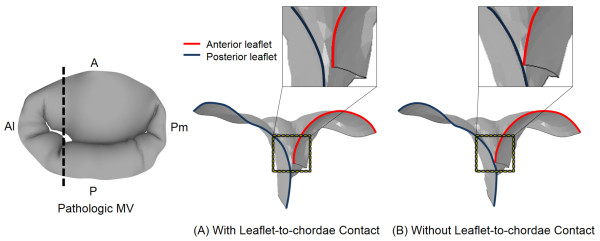
Leaflet coaptation of the pathologic MV involving MR at peak systole with and without incorporation of leaflet-to-chordae contact interaction.

Alteration in leaflet deformation due to the incorporation of leaflet-to-chordae contact interaction revealed a difference in leaflet coaptation of the pathologic MV (Figure 4). With leaflet-to-chordae contact interaction, the cross-sectional view of the leaflet deformation near the anterolateral commissural region at peak systole demonstrated incomplete coaptation between the anterior and posterior leaflets. This lack of leaflet coaptation disappeared when only leaflet-to-leaflet contact interaction was included (i.e., without leaflet-to-chordae contact). The maximum gap distance was 3.2 mm and 1.4 mm with and without leaflet-to-chordae contact interaction, respectively.

### Leaflet contact distribution

**Figure 5:**
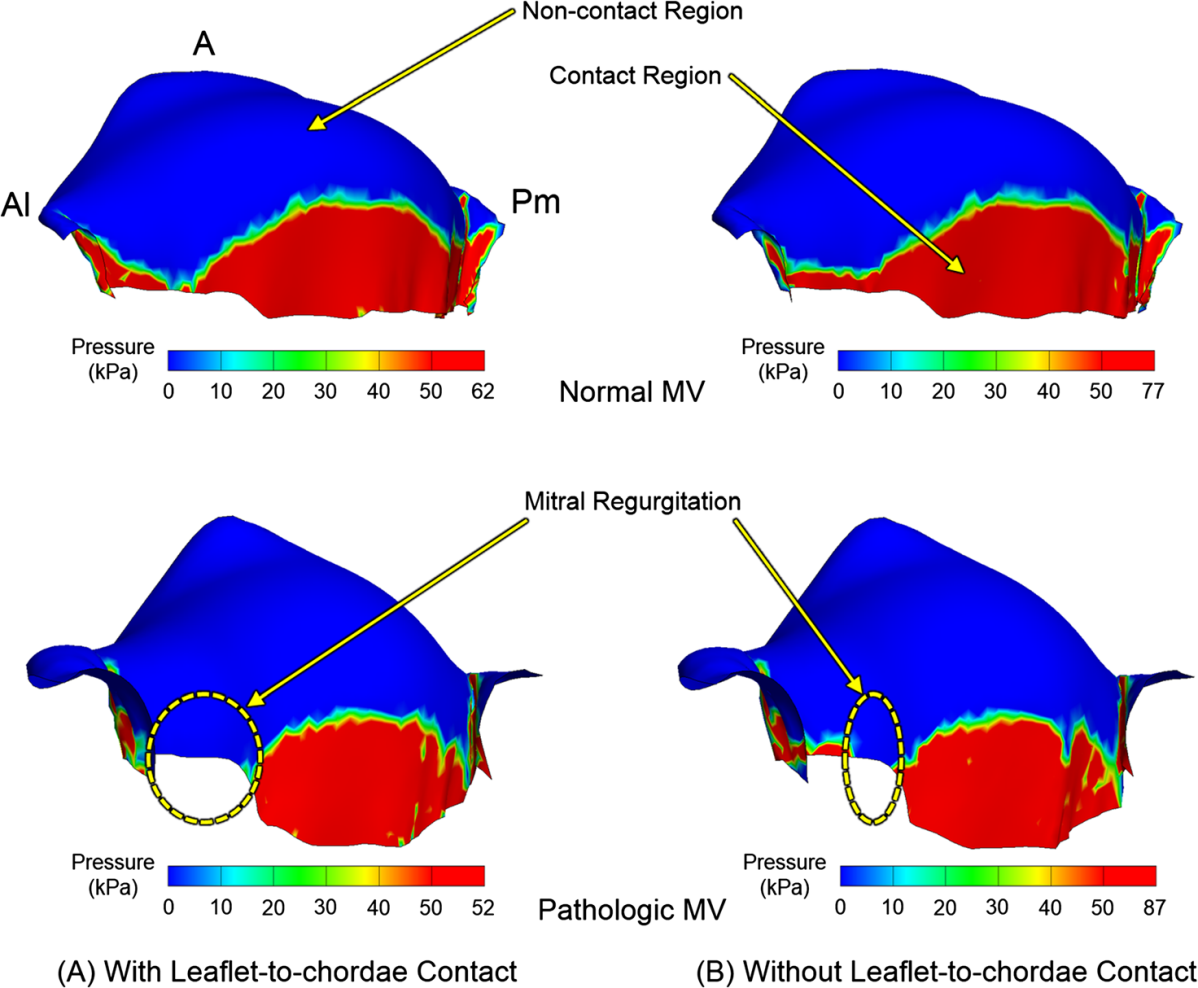
Distribution of contact pressure between the anterior and posterior MV leaflets at peak systole with and without incorporation of leaflet-to-chordae contact interaction.

The extent of leaflet coaptation, which directly correlates with the degree of MR, was determined by measuring the distribution of contact pressure between the anterior and posterior leaflets at peak systole (Figure 5). A threshold value (50 kPa) of contact pressure upon the anterior leaflet was employed to best demonstrate full contact leaflet coaptation. The maximum leaflet contact pressure decreased from 77 kPa to 62 kPa in the normal MV when leaflet-to-chordae contact interaction was incorporated. The difference of maximum leaflet contact pressure in the pathologic MV was much greater (87 kPa vs. 52 kPa) between the simulations with and without leaflet-to-chordae contact interaction.

The normal MV showed sufficient leaflet coaptation and little difference in overall leaflet contact pressure distribution between the simulations with and without incorporation of leaflet-to-chordae contact interaction. In the pathologic MV with MR, there was a large non-contact region near the anterolateral commissure, indicating a considerable occurrence of MR. The length of leaflet marginal edge with no coaptation in the anterolateral region was much larger in the simulation with leaflet-to-chordae contact interaction (6.9 mm) than for the simulation with leaflet-to-leaflet contact alone (2.6 mm). This data corresponds to the markedly enlarged size of regurgitant orifice area (10.2 mm^2^ vs. 1.4 mm^2^) when leaflet-to-chordae contact interaction was implemented. With incorporation of leaflet-to-chordae contact interaction incorporated, the size of total contact region decreased by 3.8% and 4.2% in the normal and the pathologic MVs, respectively. This indicates that leaflet-to-chordae contact interaction affects both qualitative and quantitative determination of leaflet coaptation when evaluating MV function.

### Leaflet stress distribution

**Figure 6:**
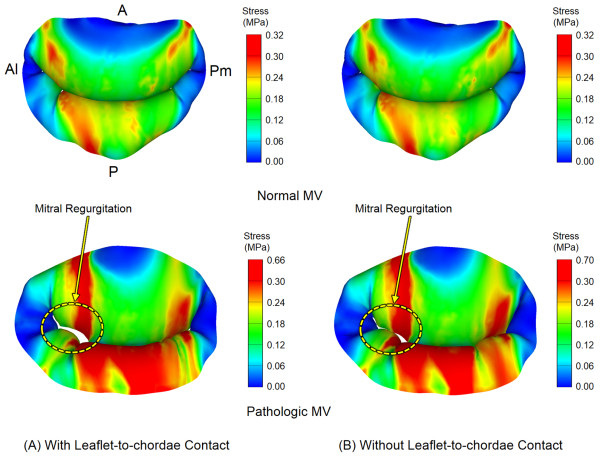
Leaflet stress distribution with and without incorporation of leaflet-to-chordae contact interaction.

The maximum principal stress distributions across the mitral annulus and leaflets at peak systole are demonstrated in Figure 6. The normal and pathologic MVs showed a difference in stress distribution pattern. The maximum stress values in the normal MV occurred near the anterior saddle horn region spreading along the circumferential direction. In the pathologic MV, a much wider range of large stress distribution was found across both leaflets spreading along the radial direction. This directional pattern of stress distribution was clearly observed around the regurgitant area of the anterior leaflet close to the anterolateral commissural region. The maximum stress values in the pathologic MV was twice larger than for the normal MV.

MV simulations with and without consideration of leaflet-to-chordae contact interaction demonstrated little difference in overall stress distribution in both normal and pathologic MVs. The maximum stress value (0.32 MPa) at peak systole was comparable in the normal MV simulations with and without leaflet-to-chordae contact interaction. However, in the pathologic MV, the maximum stress value decreased from 0.7 MPa to 0.66 MPa when leaflet-to-chordae contact interaction was incorporated. A wider range of large stress concentration was found in the regurgitant areas beside both leaflets.

## Discussion

Accurate determination of the extent and severity of MR is important as MR can cause serious pathophysiologic problems leading to left ventricular dysfunction and heart failure. The most popular modality for MR evaluation at present is color Doppler ultrasound imaging that provides semi-quantitative information of MR occurrence [26]. Computational simulations of MV function with the use of patient-specific MV geometric data obtained from clinical imaging modalities can provide additional biomechanical and functional information [17,18,27,28]. Biomechanical information such as leaflet contact pressure distribution and the extent of leaflet coaptation can directly provide quantitative information pertaining to the degree and mechanism of MR [17,18]. Therefore, it is important to correctly determine physical contact interaction between the MV apparatus components during computational MV evaluation.

In the present study, we investigated the effect of leaflet-to-chordae contact interaction on patient-specific computational MV evaluation. To the best of our knowledge, the effect of leaflet-to-chordae contact interaction on computational MV evaluation has never been fully investigated.

The greatest difference between the computational MV evaluations with and without leaflet-to-chordae contact interaction was found in the deformation of the leaflets and chordae as well as in the distribution of leaflet coaptation. Without incorporation of leaflet-to-chordae contact interaction (i.e., consideration of leaflet-to-leaflet contact only), the computational MV simulations demonstrated physically unrealistic contact interactions between the leaflets and chordae (Figure 3). The chordal structures penetrated into the leaflets as soon as contact between the two structures occurred during the systolic phase. It is clear that this non-physical geometric alteration can overestimate leaflet-to-leaflet contact and lead to inaccurate determination of leaflet coaptation (Figure 5). This increase in leaflet coaptation in computational MV evaluation can directly translate into underestimation of the severity of MR [17,18]. Several studies recently reported computational evaluation of MV function accompanied with patient-specific MV geometric data obtained from clinical imaging modalities [17,18,27,28]. The patient MVs in these studies demonstrated a high degree of asymmetric structural characteristics in the mitral annulus, leaflets and chordae distribution. The present study demonstrates that a disregard of leaflet-to-chordae contact interaction can affect computational evaluation of MV function, particularly in pathologic MVs involving considerable MR.

Stress distribution was evaluated for the normal and pathologic MVs to compare the effect of leaflet-to-chordae contact interaction on alteration of biomechanical characteristics of the MV apparatus structure during valve function (Figure 6). The pathologic MV revealed an apparent stress distribution pattern along the radial direction across both the leaflets while the normal MV showed a relatively uniform stress distribution. We speculate that this particular stress distribution pattern in the pathologic MV was induced by the dilated mitral annulus and the increased tensile stresses in the adjacent tightened chordae in the region where MR occurred. Incorporation of leaflet-to-chordae contact interaction revealed a relatively small difference in the overall pattern and magnitude of stress distribution compared to those without the contact interaction. This may be the reason why previous computational MV evaluation studies did not pay attention to leaflet-to-chordae contact interaction, and described little details on contact interaction modeling between the interrelated components of the MV apparatus [19,21,29]. We found that the difference in stress distribution may be negligible during valve function in normal MVs regardless of clear incorporation of leaflet-to-chordae contact interaction. However, there is great potential to generate a considerable error in determining the maximum stress concentration and stress distribution in pathologic MVs when leaflet-to-chordae contact interaction is not correctly incorporated in the computational simulation.

Although the present study has demonstrated the effect of contact interactions between the MV apparatus components on computational MV evaluation, the simulation results should be carefully interpreted prior to defining MV pathophysiology. Although we demonstrated computational MV evaluation using two patient data (one normal and one pathologic MVs), the physical influence of leaflet-to-chordae contact interaction should be effective in any types of MV simulation studies. We utilized previously published material characteristic data, employed geometric and physiologic parameters such as leaflet thickness and tissue density, and assumed homogeneous thickness and material behavior of the MV tissue. Although different types of tissue material behavior may affect computational MV simulation outcomes, it would not change the primary findings of this study in terms of the physical effect of leaflet-to-chordae contact interaction. Our computational simulation data was obtained purely by structural analysis, i.e. dynamic FE simulation incorporated with physiologic damping conditions. The latter implies that blood fluid effects on normal and shear pressure distribution over the MV leaflets and chordae were not fully incorporated in the computational simulations. Notwithstanding these limitations, the effect of leaflet-to-chordae contact interaction on computational MV evaluation presented in this study should be valid in any types of MV simulation studies.

## Conclusions

We have successfully demonstrated the effect of leaflet-to-chordae contact interaction on determining leaflet coaptation in computational dynamic MV evaluation. Two types of patient-specific MV geometric data were analyzed to assess the difference of this modeling effect in normal and pathologic MVs. We found that physically realistic contact interactions between the leaflets and chordae should be considered to accurately quantitate leaflet coaptation for MV simulation. Computational evaluation of MV function that allows precise quantitation of leaflet coaptation has great potential to better quantitate the severity of MR. Improved MR quantitation using this computational simulation combined with patient-specific 3D echocardiography can help us to better diagnose and treat MV pathology.

## Abbreviations

2D: Two-dimensional; 3D: Three-dimensional; FE: Finite element; MR: Mitral regurgitation; MV: Mitral valve; TEE: Transesophageal echocardiography.

## Competing interests

The authors declare that they have no competing interests.

## Authors’ contributions

YR carried out the algorithm design and implementation, and drafted the manuscript. DDM participated in the design of the study and coordination. HK conceived of the study, participated in the design of the study, and contributed to discussions and suggestions to complete the manuscript. All authors read and approved the final manuscript.

## References

[B1] Ho SY Anatomy of the mitral valve Heart 2002 88 Suppl 4 v5 v10 10.1136/heart.88.suppl_4.iv5 PMC1876279 12369589

[B2] Silbiger JJ Bazaz R Contemporary insights into the functional anatomy of the mitral valve Am Heart J 2009 158 6 887 895 10.1016/j.ahj.2009.10.014 19958853

[B3] Turi ZG Cardiology patient page: mitral valve disease Circulation 2004 109 6 e38 e41 10.1161/01.CIR.0000115202.33689.2C 14970121

[B4] Muresian H Diena M Cerin G Filipoiu F The mitral valve: new insights into the clinical anatomy J Clin Med 2006 1 4 80 87

[B5] Fabricius AM Walther T Falk V Mohr FW Three-dimensional echocardiography for planning of mitral valve surgery: current applicability? Ann Thorac Surg 2004 78 2 575 578 10.1016/j.athoracsur.2003.10.031 15276524

[B6] Hung J Lang R Flachskampf F Shernan SK McCulloch ML Adams DB Thomas J Vannan M Ryan T 3D echocardiography: a review of the current status and future directions J Am Soc Echocardiogr 2007 20 3 213 233 10.1016/j.echo.2007.01.010 17336747

[B7] Aupart MR Babuty DG Guesnier L Meurisse YA Sirinelli AL Marchand MA Double valve replacement with the Carpentier-Edwards pericardial valve: 10-year results J Heart Valve Dis 1996 5 3 312 316 8793683

[B8] Prot V Haaverstad R Skallerud B Finite element analysis of the mitral apparatus: annulus shape effect and chordal force distribution Biomech Model Mechanobiol 2009 8 1 43 55 10.1007/s10237-007-0116-8 18193309

[B9] Sacks MS The biomechanical effects of fatigue on the porcine bioprosthetic heart valve J Long Term Eff Med Implants 2001 11 3–4 231 247 11921666

[B10] Sacks MS Schoen FJ Collagen fiber disruption occurs independent of calcification in clinically explanted bioprosthetic heart valves J Biomed Mater Res 2002 62 3 359 371 10.1002/jbm.10293 12209921

[B11] Chandran KB Role of computational simulations in heart valve dynamics and design of valvular prostheses Cardiovasc Eng Technol 2010 1 1 18 38 10.1007/s13239-010-0002-x 20606715 PMC2893742

[B12] Kim H Chandran KB Sacks MS Lu J An experimentally derived stress resultant shell model for heart valve dynamic simulations Ann Biomed Eng 2007 35 1 30 44 17089074 10.1007/s10439-006-9203-8

[B13] Kim H Lu J Sacks MS Chandran KB Dynamic simulation pericardial bioprosthetic heart valve function J Biomech Eng 2006 128 5 717 724 10.1115/1.2244578 16995758

[B14] Kim H Lu J Sacks MS Chandran KB Dynamic simulation of bioprosthetic heart valves using a stress resultant shell model Ann Biomed Eng 2008 36 2 262 275 10.1007/s10439-007-9409-4 18046648

[B15] Kunzelman KS Cochran RP Stress/strain characteristics of porcine mitral valve tissue: parallel versus perpendicular collagen orientation J Card Surg 1992 7 1 71 78 10.1111/j.1540-8191.1992.tb00777.x 1554980

[B16] Kunzelman KS Quick DW Cochran RP Altered collagen concentration in mitral valve leaflets: biochemical and finite element analysis Ann Thorac Surg 1998 66 6 Suppl S198 S205 9930448 10.1016/s0003-4975(98)01106-0

[B17] Rim Y Laing ST Kee P McPherson DD Kim H Evaluation of mitral valve dynamics J Am Coll Cardiol Img 2013 6 2 263 268 10.1016/j.jcmg.2012.10.017 PMC3629823 23489540

[B18] Rim Y McPherson DD Chandran KB Kim H The effect of patient-specific annular motion on dynamic simulation of mitral valve function J Biomech 2013 46 6 1104 1112 10.1016/j.jbiomech.2013.01.014 23433464 PMC3629842

[B19] Prot V Skallerud B Sommer G Holzapfel GA On modelling and analysis of healthy and pathological human mitral valves: two case studies J Mech Behav Biomed Mater 2010 3 2 167 177 10.1016/j.jmbbm.2009.05.004 20129416

[B20] Stevanella M Votta E Redaelli A Mitral valve finite element modeling: implications of tissues’ nonlinear response and annular motion J Biomech Eng 2009 131 12 121010 10.1115/1.4000107 20524733

[B21] Votta E Maisano F Bolling SF Alfieri O Montevecchi FM Redaelli A The Geoform disease-specific annuloplasty system: a finite element study Ann Thorac Surg 2007 84 1 92 101 10.1016/j.athoracsur.2007.03.040 17588392

[B22] May-Newman K Yin FC A constitutive law for mitral valve tissue J Biomech Eng 1998 120 1 38 47 10.1115/1.2834305 9675679

[B23] May-Newman K Yin FC Biaxial mechanical behavior of excised porcine mitral valve leaflets Am J Physiol 1995 269 4 Pt 2 H1319 H1327 7485564 10.1152/ajpheart.1995.269.4.H1319

[B24] Abaqus 6.11 documentation [ http://abaqus.me.chalmers.se/v6.11/books/usi/usi-link.htm]

[B25] Stefancu A Melenciuc S Budescu M Penalty based algorithms for frictional contact problems Bulletin of the Polytechnic Inst of Iasi - Construction & A 2011 61 3 119 129

[B26] Zoghbi WA Enriquez-Sarano M Foster E Grayburn PA Kraft CD Levine RA Nihoyannopoulos P Otto CM Quinones MA Rakowski H Stewart WJ Waggoner A Weissman NJ American Society of Echocardiography Recommendations for evaluation of the severity of native valvular regurgitation with two-dimensional and Doppler echocardiography J Am Soc Echocardiogr 2003 16 7 777 802 10.1016/S0894-7317(03)00335-3 12835667

[B27] Votta E Le TB Stevanella M Fusini L Caiani EG Redaelli A Sotiropoulos F Toward patient-specific simulations of cardiac valves: state-of-the-art and future directions J Biomech 2013 46 2 217 228 10.1016/j.jbiomech.2012.10.026 23174421 PMC3552085

[B28] Wang Q Sun W Finite element modeling of mitral valve dynamic deformation using patient-specific multi-slices computed tomography scans Ann Biomed Eng 2013 41 1 142 153 10.1007/s10439-012-0620-6 22805982

[B29] Xu C Jassar AS Nathan DP Eperjesi TJ Brinster CJ Levack MM Vergnat M Gorman RC Gorman JH 3rd Jackson BM Augmented mitral valve leaflet area decreases leaflet stress: a finite element simulation Ann Thorac Surg 2012 93 4 1141 1145 10.1016/j.athoracsur.2012.01.069 22397985 PMC3462015

